# Successful Coil Embolization Treatment of a Large Arterioportal Fistula: A Rare Cause of Mesenteric Ischemia

**DOI:** 10.7759/cureus.14322

**Published:** 2021-04-06

**Authors:** Karim Nasra, Alicia Heidenreich, Matthew Nasra, Erik Wolf, Denis Lincoln

**Affiliations:** 1 Radiology, Ascension Providence/Michigan State University, Southfield, USA; 2 Radiology, Robert Wood Johnson University Hospital, New Brunswick, USA

**Keywords:** arteriovenous fistula, arterioportal fistula, mesenteric ischemia, embolization, vascular injury

## Abstract

Superior mesenteric arteriovenous fistulae (SMAVF) are a rare complication from trauma or iatrogenic surgical intervention. There are less than 50 cases reported in the literature and no clear guidelines as to the best practices for diagnosis and treatment. SMAVF are often asymptomatic but can present with nonspecific abdominal symptoms ranging from nausea and vomiting to gastrointestinal bleeding and mesenteric ischemia. Symptom onset, when present, is often delayed years after the inciting event, further complicating the diagnosis. We present a case of a 71-year-old man presenting with mesenteric ischemic symptoms secondary to a large SMAVF that was successfully treated with coil embolization. We describe our approach to treatment and describe the classical imaging findings. We, then, review the current literature and management recommendations.

## Introduction

We present a case of a patient presenting with postprandial abdominal pain secondary to a giant superior mesenteric arteriovenous fistula (SMAVF). The arteriovenous connection was successfully treated with coil embolization. There were no complications. Patient symptomatology resolved by discharge, and follow-up imaging demonstrated complete thrombosis of the SMAVF.

Arteriovenous fistulae (AVF) are communications between the arterial and venous system that bypass the high-resistance arteriole system. Blood is shunted away towards the low-pressure venous system. In the mesenteric vessels, this can result in vague, nonspecific abdominal symptoms among other symptoms.

The most common causes of mesenteric AVF are iatrogenic or trauma-related. For example, an errant stitch or suture line that crosses through an artery and vein may result in the formation of an abnormal communication between the two. The inciting incident, however, may have occurred years ago, making diagnosis challenging. Furthermore, the AVF is often detected incidentally for imaging performed for other reasons. Only once other etiologies for the patient’s symptoms have been ruled out, should the AVF be treated as the cause. Due to the rarity of SMAVF, there are no clear guidelines for treatment. Management options range from surveillance, open surgery, or minimally invasive endovascular interventions.

## Case presentation

A 71-year-old man presented to the emergency department with chief complaint of cramping abdominal pain, which he rated 10 out of 10. Symptoms have been on and off for the past two months. In addition, he admitted to nausea, vomiting, and mild non-bloody diarrhea. Vital signs were within normal limits. Past medical history included hypertension, midline anterior abdominal wall hernia, and a remote small bowel obstruction for which he underwent partial small bowel resection and anastomosis 25 years ago. He had a recent colonoscopy that revealed tubular adenomas.

Pertinent positives on physical exam included abdominal tenderness to palpation without rigidity, guarding, or distension. Labs demonstrated mildly decreased hemoglobin (12.1 g/dL) and mild elevation of liver functional enzymes (total bilirubin: 1.9, direct bilirubin 0.4, alkaline phosphatase 134, AST 84, ALT 82). Lipase, white blood cell count, lactate dehydrogenase, and lactic acid were within normal limits.

**Figure 1:**
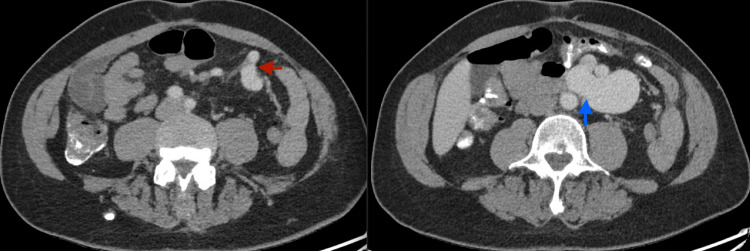
Axial contrast-enhanced CT through the abdomen showing the arterial communication at the distal branches of the superior mesenteric artery (red arrow) and the venous communication at the superior mesenteric vein (blue arrow).

**Figure 2:**
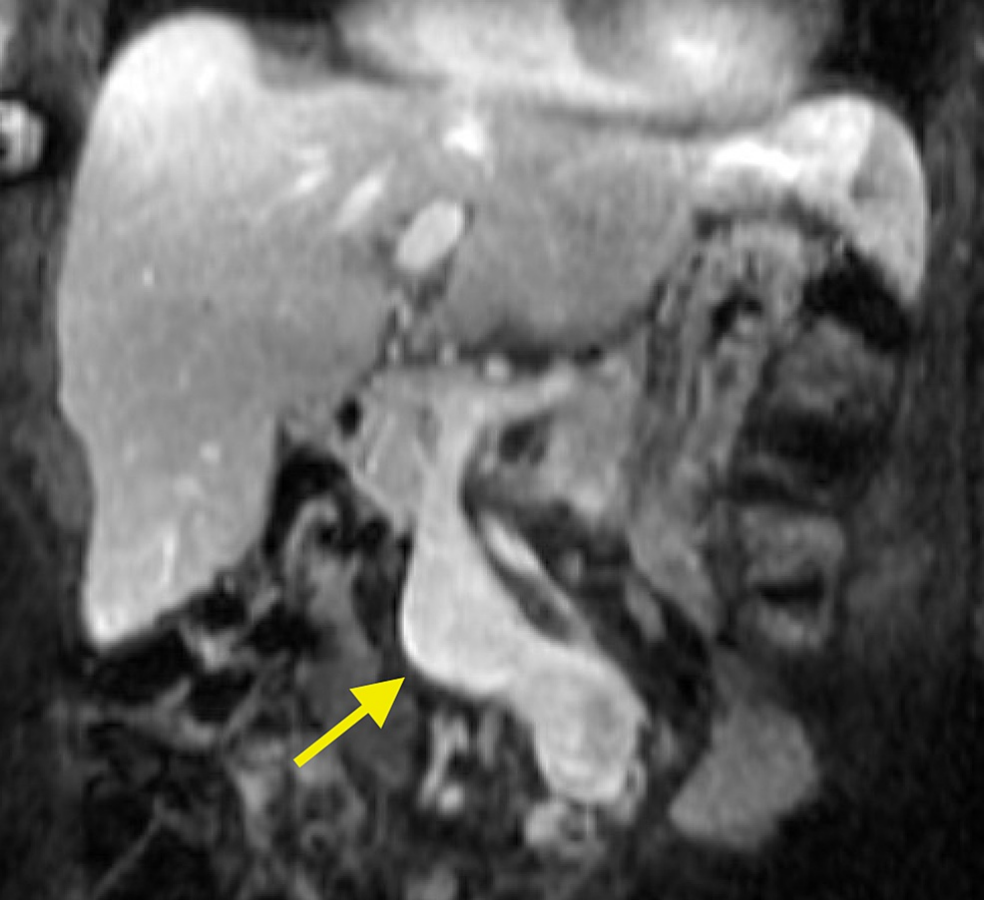
Coronal contrast-enhanced T1 weighted MRI depicting the superior mesenteric fistula in profile (yellow arrow).

The primary care team's initial concern was for an infectious or inflammatory process. A contrast-enhanced CT scan was ordered, which revealed diffuse atherosclerotic disease and a high-grade superior mesenteric artery (SMA) to superior mesenteric venous (SMV) communication with enlargement of the SMV to 3.4 cm (Figure 1). No other emergent findings were seen on the CT to explain the patient's symptomatology. The SMAVF was thought to have arisen from an insult from a remote small bowel resection. Additional MRI performed for an incidental liver lesion again demonstrated the SMAVF (Figure 2). The delayed onset of symptoms was attributed to slow growth over time until there was enough of a steal phenomenon to present with abdominal pain. Formal angiography and possible intervention were planned.

**Figure 3:**
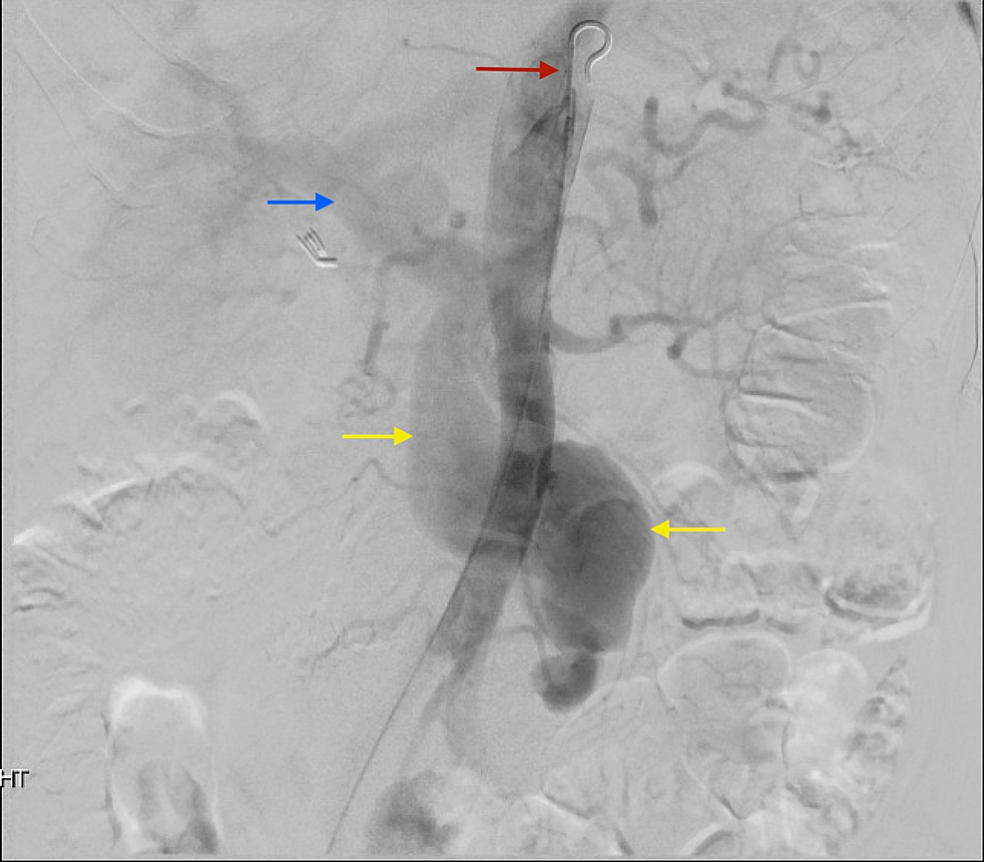
Angiography from a flush catheter in the aorta (red arrow) depicts early opacification of a large draining vein, the superior mesenteric vein (yellow arrows). Early filling of the portal vein (blue arrow) is also noted.

**Figure 4:**
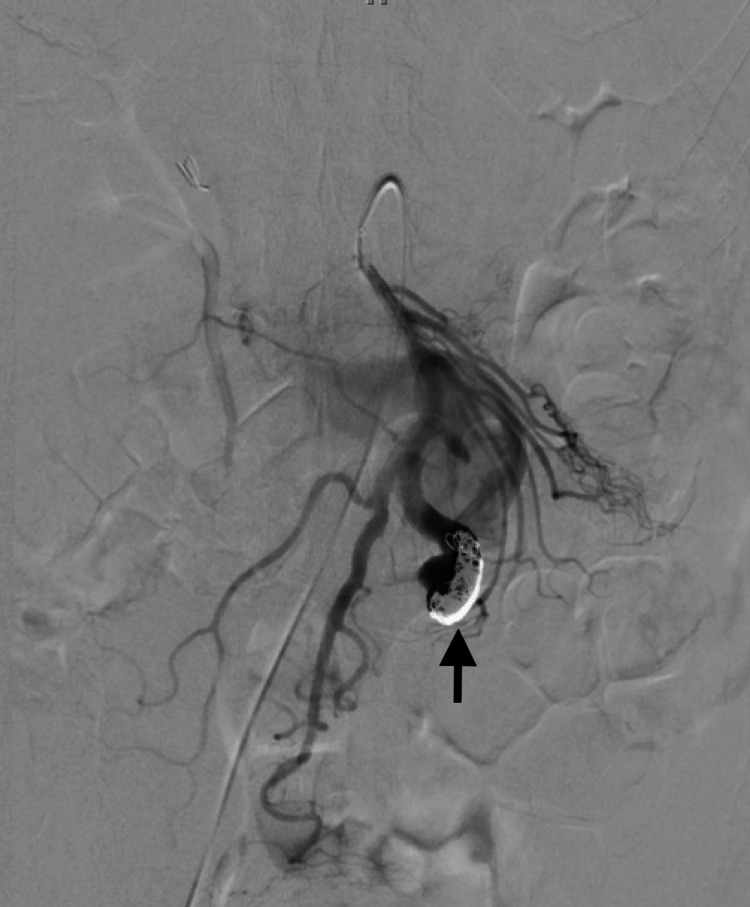
Post-embolization arteriogram from the superior mesenteric artery depicts markedly reduced opacification of the superior mesenteric arteriovenous fistula with minimal residual flow. Note the densely packed coils at the arterial inflow (black arrow).

The following day, the patient went to the angiographic suite. A 4 French micropuncture kit was used to access the right common femoral artery and upsized to a 5 French vascular sheath. Using standard catheter and guidewire technique, a 5 French Omni Flush catheter was advanced to the suprarenal abdominal aorta, and digital subtraction angiography was performed. The aortogram demonstrated normal patency of the celiac and superior mesenteric artery origins. A large, early draining vein, the SMV, was identified arising from the third-order jejunal branches (Figure 3). The SMA was then selected, and repeat DSA confirmed the selection of the SMA and provided a roadmap. The catheter was exchanged for a 6 French destination sheath at the origin of the SMA, through which a 4 French glide catheter was advanced to the distal SMA branch just proximal to the fistulous connection. Satisfactory positioning was confirmed with DSA angiography. Coils (measuring 20 mm x 20 cm, 6 mm x 20 cm, 4 mm x 7 cm, 5 mm x 11 cm) were sequentially deployed, each time followed by angiographic interrogation. Final post-embolization imaging demonstrated marked decreased flow through the SMAVF (Figure 4). All of the jejunal and ileal branches seen pre-embolization were again noted and found patent. All guidewires and catheters were removed, and hemostasis was achieved with a 6 French Angio-Seal device and manual pressure. There were no immediate or delayed complications.

**Figure 5:**
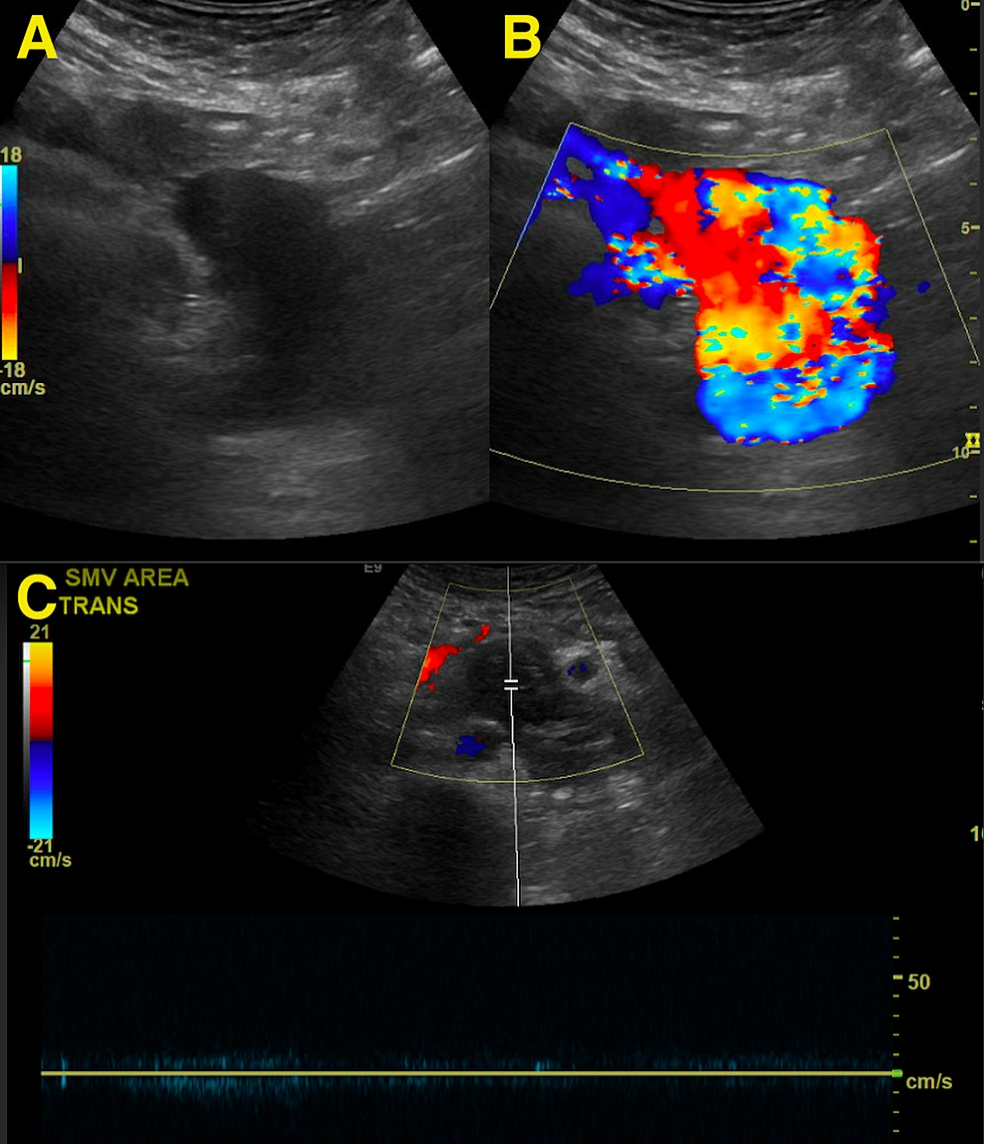
Pre-intervention gray-scale (A) and color (B) Doppler ultrasound demonstrating increased turbulent flow across the superior mesenteric arteriovenous fistula. Three-month follow-up duplex ultrasound (C) demonstrates complete thrombosis of the fistulous connection without any appreciable flow.

There was complete resolution of symptoms by the time of discharge. A follow-up ultrasound examination in three months demonstrated satisfactory thrombosis of the previously seen SMAVF without any residual flow (Figure 5).

## Discussion

SMAVF are a rare source of ischemic mesenteric complications, with less than 50 cases reported in the literature [1-3]. SMAVF may rarely be a congenital anomaly or result from visceral aneurysmal rupture. There is an association with connective tissue disease. The most common causes, however, are trauma and iatrogenic injury [4].

SMAVF are often asymptomatic, which may partially contribute to the low reported incidence. Symptom onset is often delayed and ranges in days to decades after the inciting event (if an inciting event is found) [3,4]. Symptoms are generally nonspecific at presentation. Patients may present with abdominal pain, nausea, vomiting, or diarrhea. While rare, gastrointestinal bleeding, ascites, and mesenteric ischemia are some of the more alarming presentations [5]. Complications are not limited to the gastrointestinal system. Increased venous pressure from preferential flow into the portal system may result in portal hypertension. Further, increased flow bypassing the capillary system may lead to high-output cardiac failure [6].

SMAVF is a diagnosis made primarily through imaging. Physical exam findings are generally nonspecific. The most-reported physical exam finding is an abdominal bruit from the rapid flow of high-pressure arterial blood directly into the low-pressure venous system. These are hard to distinguish from other bruits from arterial occlusive disease [1,5]. While the identification of SMAVF can be made with duplex ultrasound or cross-sectional imaging, complete characterization is best achieved with mesenteric angiography [7].

SMAVF were traditionally surgically excised or ligated, but the current preference is endovascular embolization due to comparable results and fewer complications. The mortality rate for surgical treatment of SMAVF has been reported at up to 18% [8]. Complications specific to embolization include coil migration and distal bowel necrosis. The risk is magnified in larger fistulae requiring discretion when deciding to deploy every coil.

Coils are metallic vascular-occluding devices that assume a coiled-configuration when deployed in the target vessel. Multiple coils are densely packed to achieve vessel occlusion. As the vessel is packed more tightly, however, the higher the chance that any additional coil may prolapse into a nontarget vessel due to incorrect sizing or catheter kickback. Detachable, rather than pushable, coils alleviate some but not all of the associated risks. In a high-flow, arteriovenous fistula, distal migration through the venous outflow is another potential complication. A flow-reducing device such as a vascular plug can help secure the coils if needed. Immediately post-coiling, slow blood flow is expected, and one should wait three to five minutes before reassessing residual flow within the vessel [9].

## Conclusions

Symptomatic SMAVF are quite rare, so there is limited data set to define optimal management. There is no formal recommendation for treating arterioportal fistulae with management dictated by operator familiarity and comfort. While endovascular options traditionally include coiling, there have been reports of success employing covered stents. There has been a global shift to minimally invasive endovascular techniques, and these techniques show comparable results to open surgical repair with less overall morbidity.
